# The level and determinants of knowledge and attitude towards nutrition among pregnant women in Minjar Shenkora district, Ethiopia

**DOI:** 10.1017/jns.2024.19

**Published:** 2024-07-30

**Authors:** Meron Demisew, Habtamu Fekadu Gemede, Kassahun Ayele

**Affiliations:** 1 Department of Food and Nutritional Science, Wollega Uiversity Shambu Campus, Shambu, Ethiopia; 2 Department of Food Technology and Nutrition, Wollega University, Nekemte, Ethiopia

**Keywords:** Attitude, Ethiopia, Knowledge, Minjar Shenkora, Nutrition, Pregnant women

## Abstract

Maternal knowledge and attitudes towards nutrition are essential for achieving healthy dietary behaviours for a positive pregnancy outcome. Therefore, this study aimed to assess the level and determinants of knowledge and attitudes towards nutrition among pregnant women in the Minjar Shenkora district, Ethiopia. Institution-based cross-sectional study was conducted between June and August 2021 among 334 pregnant women who were selected using a systematic random sampling technique. Data were collected using a semi-structured questionnaire and analysed using SPSS version 21. Bivariate and multivariate logistic regression analyses were used to identify the determinants of nutritional knowledge and attitudes. P < 0.05, with a 95% CI was used to declare statistical significance. Overall, 69.2% and 44.0% of the pregnant women were not knowledgeable and had an unfavourable attitude toward nutrition, respectively. The educational status of pregnant women (P < 0.01) and access to nutritional information (P < 0.01) were significantly associated with nutritional knowledge, while the educational status of pregnant women (P < 0.01) was associated with nutritional attitude. Respondents’ knowledge of and attitudes toward nutrition were low. Pregnant women’s educational levels and access to nutritional information are strongly related to their nutritional knowledge. Likewise, the educational status of the pregnant women was significantly associated with their nutritional attitudes. Therefore, education and counselling on maternal nutrition are needed to improve healthy maternal nutrition tips in the study area.

## Introduction

Pregnancy is an anabolic process, and pregnant women have a higher demand than non-pregnant women, possibly because of the growing number of uterine cells.^(1,2)^ It is a critical period in the life cycle when additional nutrients are required to meet the metabolic and physiological demands as well as the increased needs of the growing foetus. Pregnant women are more vulnerable to malnutrition owing to the high demand for nutrients to deposit energy in the form of new tissue, the growth of existing maternal tissues such as the breast and uterus, and the increased energy requirements for tissue synthesis.^(3,4)^ Adequate nutrition is essential for the health and reproductive performance of women as well as the health, survival, and development of children.^(5–7)^ However, maternal nutritional deficiency is a global public health problem that contributes to the death of 3.5 million women annually, mainly in low- and middle-income countries.^(8,9)^ Africa has significantly higher undernutrition in pregnancy, and sub-Saharan Africa is the most affected region.^(10–12)^ Ethiopia is the country where the undernutrition problem is still among the major health problems, especially among children and mothers, with a higher prevalence.^(13–17)^

In Ethiopia, nearly one in five pregnant women are malnourished in rural areas and are approximately 68% more likely to be malnourished than urban women.^(18)^ To ensure positive pregnancy outcomes, pregnant women should have adequate nutritional knowledge and attitude. Malnutrition and other nutrition-related complications are immediately caused by a lack of knowledge and attitude.^(19,20)^ Nutritional knowledge is predictive of changes in dietary habits, and health advice encourages pregnant women to increase their food intake.^(21,22)^ Nutritional knowledge during pregnancy is also important for increasing maternal awareness of proper nutrition.^(23)^ Nutritional attitude also refers to the affective feelings of expectant mothers who like or dislike prenatal services. As a result, pregnant women’s personal experiences with prenatal services can be favourable or unfavourable.^(24,25)^ Women should be made aware of the effects of healthy nutritional attitudes, either before conception or as early as possible during pregnancy. Good knowledge and a favourable attitude toward optimal nutrition are essential elements to ensure a positive pregnancy outcome and are integral to achieving healthy dietary behaviours, which then improve the quality of the diet.^(26,27)^ This is true for pregnant women, as good maternal nutrition is one of the best ways to ensure maternal and foetal well-being.

Good knowledge of maternal nutrition and a favourable attitude toward pregnancy is necessary to ensure positive pregnancy outcomes.^(22,28)^ Women’s nutrition knowledge and attitudes before pregnancy are also important in raising maternal awareness about the importance of good nutrition for both the mother and healthy foetal development. Limited knowledge and an unfavourable attitude towards nutrition influence dietary practices and are, thus, potential determinants of pregnancy outcomes, including maternal mortality, infant mortality, and low birth weight in infants.^(22,27,28)^ The World Health Organization (WHO) has approved antenatal nutrition education for women to reduce the birth of underweight infants and prevent maternal complications. The WHO also implements a nutrition education and counselling strategy focusing on increasing the variety and quantity of foods consumed, promoting sufficient and balanced dietary intake, and encouraging the regular use of fortified foods.^(29)^ Ethiopia’s Ministry of Health (MoH) has used different strategies, such as nutrition education via social media.^(30)^ Similarly, health extension workers have been working to address women’s lack of dietary knowledge and attitude. However, approximately 47% of women lack knowledge of balanced and diversified diets during pregnancy.^(31)^ Similarly, only 27% and 48.4% of pregnant women were knowledgeable and had a favourable attitude toward the need to eat a varied and balanced diet, respectively.^(32)^ Pregnant women’s knowledge of and attitudes toward nutrition are influenced by several factors. These include the occupational status of respondents, the mother’s educational level, her monthly income, her ANC visits, the size of her family, and her marital status among pregnant women in Ethiopia.^(31,33–35)^

Even though few studies on nutrition knowledge and attitudes have been done in Ethiopia, the studies indicate inconsistency in the proportion and contributing variables of pregnant women’s knowledge and attitudes toward nutrition.^(31,32,36,37)^ Although factors such as educational status, occupation, family size, and monthly income have been associated with maternal nutrition knowledge and attitude, some local and environmental determinants such as place of residence, access to nutrition information, and pregnancy conditions are variables that were not statistically identified. In addition, previous studies on maternal nutritional knowledge and attitude are not sufficient to improve and provide recommendations on maternal nutrition counselling and education programmes because the studies focused on communities’ urban areas, regions, and zone levels and did not consider rural settings. Accordingly, additional findings are needed, especially in rural communities, to identify nutritional knowledge and attitude, as well as the contributing factors to implementing evidence-based maternal nutrition counselling and education programmes, and to strengthen the few initiated programmes in the country. Despite research efforts, no study has documented the level and determinants of knowledge and attitudes towards nutrition among pregnant women in the study area. Therefore, this study aimed to assess the level and factors of knowledge and attitude towards nutrition among pregnant women in the Minjar Shenkora district, Ethiopia.

## Methods

### Study area, design, and period

This institutional-based cross-sectional study was conducted from June to August 2021 in public health centres in the Minjar Shenkora district of Ethiopia. The district is located at the southern end of the North Shewa Zone and is bordered to the east, south, and west by the Oromia region, to the northwest by Hegere Mariam, and to the northeast by Berehet Woreda. It is an administrative woreda of the North Shewa Zone, Amhara National Regional State. Arerti is the administrative centre of Minjar Shenkora Woreda, which is located 135 km from Addis Abeba, Ethiopia’s capital city. According to the 2007 population census report, the district has a total population of 128879 people, comprising 66918 men and 61.961 women. Six public health centres in the district provide antenatal services to the public, including Arerti Hospital. According to a health centre report, an average of 515 pregnant women follow antenatal care services within two months in the district.

### Experimental methods

The study was conducted in accordance with the guidelines of the Declaration of Helsinki, and all procedures involving human subjects/patients were approved by the Wollega University Ethical Review Committee (WU 486/2021). Written informed consent was obtained from all subjects.

#### Source and study population

The source population of this study was pregnant women attending antenatal care services in the public health centres of the Minjar Shenkora District. The study subjects consisted of systematically selected pregnant women who were attending ANC follow ups in public health centres in Minjar Shenkora District during the study period.

#### Inclusion criteria and exclusion criteria

Pregnant women who visited public health centres for antenatal care service follow up and lived in the district for at least six months in the district were included in the study. Pregnant women who were seriously ill during the data collection period, lived below six months in the study area, could not listen or speak, and were referred from other health centres were excluded from the study.

### Sample size determination

The required sample size for this study was calculated using a single population proportion formula, considering a previous finding of 27% with a 95% confidence and 5 % margin error.^(32)^ The number of samples was calculated using the following formula: 
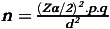


**N** = the sample size. **P** = proportion of nutritional knowledge (27%) and **d** = margin of error (required precision), which was assumed to be 5%. **Z =** upper percentile of the normal distribution. Considering a nonresponse rate of 10%, the final study sample size was 334.

### Sampling techniques

The calculated sample size was proportionally allocated to six public health centres, Bolo Health Center, Balchi Health Center, Dire Health Center, Kristos Semra Health Center, and Arerti Health Center, including Arerti Hospital, located in Minjar Shenkora District, based on the average number of clients in the respective antenatal care units before the study period. The average number of participants who visited the ANC health facility daily through data collection was predicted primarily based on the previous two months’ daily client flow in the clinic, which was obtained by referring to client registration books or records before data collection. The average number of flow records for ANC service in health centres for the previous two months total of those who visited was 515. The sampling interval was calculated using the equation (k = N/n = 515/334 = 2). The first participant women were randomly selected using the lottery method, and every second woman was selected using a simple random sampling technique until the required sample size was attained.

### Data collection tools and procedure

Data were collected using a semi-structured questionnaire adapted from different previous literature.^(31,33,35)^ The questionnaire consisted of questions on sociodemographic characteristics, obstetric and health-related characteristics, nutritional knowledge, and nutritional attitude. Data were collected by three trained diploma-holding enumerators and one bachelor’s holder assigned as a supervisor.

### Data quality control

The questionnaire was carefully translated into Amharic and then back to English to ensure consistency. To preserve data quality, the questionnaires were pre-tested on 5% of pregnant women, and corrections and modifications were made based on the gaps identified during the interview. Data collectors and supervisors were trained in the study’s background, objectives, tools, and ethical procedures. Training for data collectors emphasised the importance of keeping the study participant information confidential. Furthermore, supervisors had to check how the data collectors performed their tasks, including ethical procedures, and verify the completion of questionnaires during the data collection process.

### Data processing and analysis

The collected data were edited, coded, cleaned, and checked for consistency and completeness. The data were put *epi* data Version 3.1 software and then exported to the Statistical Package for Social Science (SPSS) Version 21 for analysis. The data were changed to analyse the use of SPSS Model 21 software. Descriptive statistics was used to describe the characteristics of the study population. Bivariate logistic regression at a 95 % confidence interval (CI) was used to assess the predict association between the dependent and independent variables. Variables with a P < 0.2 in the bivariate logistic regression were inserted at the same time in the multivariate logistic regression to control for possible confounding. Multivariate logistic regression analysis with an adjusted odds ratio at 95 %CI was performed to assess the determinants of knowledge and attitude toward nutrition, and associations were declared significant at P < 0.05 to declare the results statistically significant. Hosmer-Lemeshow examined what was used to test the model’s fitness.

### Operational definitions

Knowledge refers to an individual’s understanding of nutrition, including their intellectual ability to remember and recall food- and nutrition-related terminology, specific pieces of information, and facts.^(38,39)^

Knowledgeable: The respondent answers ≥ 70% (out of 100%) of the knowledge questions.^(36)^

Not knowledgeable: The respondent answered < 70% (out of 100%) to the knowledge questions.^(40)^

Attitudes are emotional, motivational, perceptive, and cognitive beliefs that positively or negatively influence an individual’s behaviour or practice.^(41,42)^

Favourable attitude: if respondents score attitude questions ≥ mean^(32,37)^

Unfavourable Attitude: if respondents score for attitude questions < mean^(32,37)^

### Ethical approval and consent to participate

This study was conducted in accordance with the Declaration of Helsinki guidelines. The Wollega University Ethical Review Committee approved this study (WU /453/2021). A formal permission letter was sent to the administrators of the Minjar Woreda Health Office and to each of the health centres that also approved the study. Informed consent was obtained from each participant at the time of the data collection. All procedures were performed according to the relevant rules and regulations described in the methods section. The data collectors informed clients that they had the right to cancel or refuse participation in the study, and client responses were treated confidentially to uphold this.

## Results

### Socio-demographic characteristics of pregnant women

A total of 334 women were interviewed, resulting in a response rate of 100 %. More than half of the 190 respondents (56.9 %) were aged 25–34 years. The majority of the respondents, 267 (79.9%), were married. A total of 247 (74%) respondents were followers of the Orthodox Tewhido Church. Approximately 281 (84.1%) participants were Amhara in their ethnic group (Table 1).

**Table 1.** Socio-demographic characteristics of pregnant women in Minjar Shenkora district, Ethiopia, 2021 (n = 334)

Variables	Categories	Frequency (%)
Age	18–24	107 (32)
	25–34	190 (56.9)
	35–44	37 (11.1)
Marital status	Single	29 (8.7)
	Married	267 (79.9)
	Divorced	24 (7.2)
	Widowed	14 (4.2)
Religion	Christian	247 (74)
	Muslim	65 (19.5)
	Others^a^	22 (6.6)
Ethnicity	Amhara	281 (84.1)
	Others^b^	53 (15.9)
Place of residence	Rural	121 (36.2)
	Urban	213 (63.8)
Family size	One	12 (3.6)
	Two	90 (26.9)
	Three	111 (33.2)
	Four	85 (25.4)
	Five and above	36 (10.8)
Educational women	No formal education and primary school	185 (55.4)
	9–12 class	90 (26.6)
	College and above	59 (17.7)
Educational of husband	No formal education & primary school	116 (43.4)
	9–12 class	77 (28.8)
	College and above	74 (27.7)
Occupation women	Housewife	169 (50.6)
	Private sector	96 (28.7)
	Governmental	45 (13.5)
	Others^c^	24 (7.2)
Occupation of husband	Private sector	78 (29.2)
	Governmental	68 (25.5)
	Farmer	98 (36.7)
	Others^c^	23 (8.6)
Monthly income	<2000 ETB	183 (54.8)
	≥ 2000 ETB	151 (45.2)

a Protestants and Catholics.

b Oromo, Argoba, and Tigre.

c Daily labour and Merchant.

### Obstetrics and health characteristics of pregnant women

The majority of the respondents, 215 (64.4%), were multigravida and 186 (55.7%) were multiparous. More than half (55.4%) of the participants were in their third trimester of pregnancy. One-third, 120 (34.9%) of the women had four or more antenatal visits during their current pregnancy, and 137 (41%) accessed pregnancy-related nutritional information from different sources (Table 2).

**Table 2.** Obstetrics and health characteristics of pregnant women in Minjar Shenkora district, Ethiopia, 2021 (n = 334)

Variables	Categories	Frequency (%)
Gravida	Primigravida	119 (35.6)
Multigravida	Multigravida	215 (64.4)
Parity	Primipara	148 (44.3)
Multipara	Multipara	186 (55.7)
Trimester	First trimester	40 (12.0)
Second trimester	Second trimester	109 (32.6)
Third trimester	Third trimester	185 (55.4)
Previous delivery	Normal	293 (87.7)
	Abnormal	41 (12.2)
Associated disease	Yes	97 (29.0)
No	No	237 (71.0)
Intake of iron folate supplement	Yes	190 (56.9)
	No	144 (43.1)
Antenatal follow up	Once	64 (19.2)
	Twice	68 (20.4)
	Three times	82 (24.6)
	Four and above	120 (35.9)
Nutritional information	Yes	137 (41)
	No	197 (59)

### Level of knowledge towards nutrition among pregnant women

In this study, only 103 (30.8%) respondents were knowledgeable, while the remaining 231 (69.2%) were not. About 222 (66.5%), 140 (41.9%), and 164 (49.1%) pregnant women were aware of a balanced diet, the importance of iron, and how pregnant women’s diets differed from those of non-pregnant women (Table 3).

**Table 3.** Level of knowledge towards nutrition among pregnant women in Minjar Shenkora district, Ethiopia, 2021 (n = 334)

Variables	Know frequency (%)	Don’t know frequency (%)
Balanced diet	222 (66.5)	112 (33.5)
Pregnant diet differs from non-pregnant diet	164 (49.1)	170 (50.9)
Component of balanced diet	183 (54.8)	151 (45.2)
Source of protein	141 (42.2)	193 (57.8)
Important of protein for mother and foetus	132 (39.5)	202 (60.5)
Source of vitamin	115 (34.4)	219 (65.6)
Source of calcium	102 (30.5)	232 (69.5)
Important of milk and milk product	119 (35.6)	215 (64.4)
Micronutrient supplements for pregnant women	94 (28.1)	240 (71.9)
Health benefit of folic acid for baby	88 (26.3)	246 (73.7)
Source of iron	161(48.2)	173 (51.8)
Important of iron	140 (41.9)	194 (58.1)
Important of birth spacing	205 (61.4)	129 (38.6)
The danger of malnutrition for pregnant	147 (44)	187 (56)
The danger of malnutrition for babies	156 (46.7)	178 (53.3)
Level of nutritional knowledge		
Knowledgeable	103 (30.8%)	
Not knowledge	231 (69.2%)	

### Determinant factors of knowledge towards nutrition among pregnant women

In the bivariate analysis, pregnant women’s education, place of residence, monthly average income, and inaccessible nutritional information predicted associations with nutritional knowledge. However, in a multivariate analysis, educational status and access to nutritional information were significantly associated with nutritional knowledge during pregnancy. Pregnant women with an educational level of college or above were six times more likely to be knowledgeable about nutrition during pregnancy than those with no formal education (P < 0.01). Likewise, pregnant women with access to nutrition information were twice as likely to be knowledgeable as women without access to nutrition education during their pregnancy (Table 4).

**Table 4.** Bivariate and multivariate analysis of associated with knowledge towards nutrition among pregnant women in Minjar Shenkora district, Ethiopia, 2021 (n = 334)

		Nutritional knowledge		95% CI		
Variables		Knowledgeable	Not knowledgeable	COR	AOR	P value
Residence	Rural^a^	21 (17.5%)	99 (82.5%)	1		
	Urban	82 (38.3%)	132 (61.7%)	2.92 (1.69, 5.05)	1.11 (0.46, 2.67)	ns
Women’s education	No formal and primary school	21 (11.4%)	164 (88.6%)	1	1	
	9–12 class	48 (53.3%)	42 (47.7%)	8.92 (4.82, 16.50)	0.78 (0.29, 2.14)	
	College and above	34 (57.6%)	25 (42.4%)	10.62 (5.33, 21.12)	6.92 (2.13, 22.42)^b^	0.001
Husband education	No formal and primary school	20 (17.3%)	96 (82.7%)	1	1	
	9–12 class	30 (38.9%	47 (61.1%)	3.06 (1.57, 5.95)	0.85 (0.33, 2.19)	
	College and above	35 (47.3%)	39 (53.7%)	4.30 (2.21, 8.36)	1.06 (0.36, 3.11)	
Women’s occupation	Housewife^a^	36 (21.3%)	133 (78.7%)	1	1	
	Private sector	31 (32.3%)	65 (57.7%)	1.76 (1.00, 3.09)	1.02 (0.15, 6.75)	
	Government sector	31 (68.9%)	14 (31.1%)	8.18 (3.94, 16.98)	0.89 (0.12, 6.37)	
	Others	5 (20.8%)	19 (79.2%)	0.97 (0.34, 2.78)	0.63 (0.08, 4.94)	
Husband occupation	Private^a^ sector	27(34.6%)	51(65.4%)	1	1	
	Governmental	38 (55.9%)	30 (41.1%)	2.39 (1.22, 4.66)	0.92 (0.20, 4.21)	
	Farmer	17 (17.4%)	81 (82.6%)	0.39 (0.19, 0.79)	0.36(0.07, 1.74)	
	Others	3 (13%)	20 (87%)	0.28 (0.77, 1.04)	0.92 (0.02, 4.21)	
Average monthly income	<2000 ETB^a^	35 (19.1%)	148 (80.9%)		1	
	≥ 2000 ETB	68 (45%)	83 (55%)	3.46 (2.12, 5.64)	0.85 (0.39, 1.86)	ns
Gravidity	Primigravida^a^	50 (42%)	69 (58%)	1	1	
	Multigravida	53 (24.7%)	162 (75.3%)	0.45 (0.28, 0.72)	0.64 (0.19, 2.15)	
Parity	Primipara	43 (23.1%)	143 (76.9 %)		1	
	Multipara	60 (40.5%)	88 (59.5%)	0.44 (0.27, 0.70)	0.62 (0.19, 2.04)	
Nutritional information	No^a^	36 (18%)	163 (82%)	1	1	
	Yes	67 (49.6%)	68 (50.4%)	4.46 (2.72, 7.31)	2.73 (1.39, 5.37)^b^	<0.001

AOR, adjusted odds ratio; COR, crude odds ratio; ETB, Ethiopian Birr; ns, No significance.

a Reference categories.

b Significant.

### Level of attitude towards nutrition among pregnant women

One hundred eighty-seven (56%) respondents had a favourable attitude, and the remaining 147 (44%) had an unfavourable attitude towards nutrition. Approximately 188 (56.3%), 179 (53.6%), and 196 (58.7%) participants favoured eating more protein, testing milk and milk products, and testing meat and other iron-rich foods during pregnancy, respectively. However, 115 (34.4%), 107 (32.1%), and 110 (32.9%) pregnant women were unsure whether to eat more food during pregnancy, drink milk during pregnancy, or prepare meals with iron-rich foods during pregnancy, respectively (Table 5).

**Table 5.** Level of attitude towards nutrition among pregnant women in Minjar Shenkora district, Ethiopia, 2021 (n = 334)

Variables	Good Frequency (%)	Not sure Frequency (%)	Not good Frequency (%)
Eat more food during pregnancy	131 (39.2)	115 (34.4)	88 (26.3)
Eat more carbohydrate during pregnancy	116 (34.4)	106 (31.7)	112 33.5)
Eat more protein or beans during pregnancy	188 (56.3)	77 (23)	69 (20.7)
Eat more milk during pregnancy	140 (41.7)	107 (32.1)	87 (26)
To prepare meals with iron-rich food	137 (41)	110 (32.9)	87 (26)
Test of meat and other iron-rich food	196 (58.7)	59 (17.6)	79 (23.7)
Test of omega3-rich food; olive oil, fish	103 (28.5)	101 (30.2)	130 (38.9)
Test of milk and milk product	179 (53.6)	52 (15.6)	103 (30.8)
Prepare meals with iodine salt	215 (64.4)	102 (30.5)	17 (5.1)
Level of nutritional attitude			
Favourable attitude	187 (56 %)		
Unfavourable attitude	147 (44%)		

### Determinant factors of attitude towards nutrition among pregnant women

The results of bivariate analysis showed that the educational status of pregnant women, husbands’ educational status, pregnant women’s occupation, and inaccessibility of nutrition information predicted nutritional attitudes. However, in the multivariate analysis, the educational level of women was significantly associated with their attitude towards nutrition during pregnancy. Pregnant women whose educational level was college and above were four times more likely to have a favourable attitude towards nutrition than those who had no formal education during pregnancy (Table 6).

**Table 6.** Bivariate and multivariate analysis of associated attitudes towards nutrition among pregnant women in Minjar Shenkora district, Ethiopia, 2021 (n = 334)

		Nutritional attitude		95%CI		
Variables		favourable	unfavourable	COR	AOR	P value
Women’s education	No formal and primary school^a^	71 (33%)	144 (67%)	1	1	
	9–12 class	69 (76.7%)	21 (23.3%)	5.27 (2.98, 9.34)	0.81 (0.27, 2.44)	
	College and above	47 (79.7%)	12 (20.3%)	6.28 (3.12, 12.66)	4.26 (1.47, 12.34)^b^	<0.001
Husband education	No formal and primary school^a^	53(45.7%)	63 (54.3%)	1	1	
	9–12 class	51 (62.2%)	26 (37.8%)	2.19 (1.20, 4.00)	0.91 (0.37, 2.20)	
	College and above	48 (64.9%)	26 (35.1%)	2.33(1.28–4.23)	1.44 (0.58, 3.55)	
Women’s occupation	Housewife^a^	84 (49.7%)	85 (50.3%)	1	1	
	Private sector	54 (56.3%)	42 (43.7%)	1.30 (0.78, 2.15)	1.56 (0.42, 6.29)	
	Governmental	38 (84.4%)	7 (15.6%)	5.49 (2.32–12.99)	1.63 (0.37, 6.48)	
	Others	11 (45.8%)	13 (54.2%)	0.85 (0.36, 2.01)	0.36 (0.05, 2.52)	ns
Husband occupation	Private sector^a^	48 (61.5%)	30 (38.5%)	1	1	
	Governmental	46 (67.6%)	22 (32.4%)	1.30 (0.66, 2.58)	1.18 (0.35, 3.94)	ns
	Farmer	48 (49%)	50 (51%)	0.60 (0.32, 1.09)	0.76 (0.27, 2.13)	
	Others	10 (43.5%)	13 (56.5)	0.48 (0.18, 1.23)	0.76 (0.35, 2.21)	
Nutritional information	No^a^	96 (48.2%)	103 (51.8%)	1	1	
	Yes	91 (67.4%)	44 (32.6%)	2.22 (1.40, 3.49)	1.32 (0.73, 2.41)	ns

AOR, adjusted odds ratio; COR, crude odds ratio; ns, No significance.

a Reference categories.

b Significant.

## Discussion

Nutritional knowledge and attitudes influence the quality of food consumed and healthy food choices. Limited nutrition knowledge and unfavourable attitudes influence eating habits and are thus possible predictors of pregnancy outcomes such as maternal mortality, infant mortality, and low newborn weight in infants. It is critical to identify the level and determinants of knowledge of and attitudes toward nutrition. Therefore, this study assessed the levels and determinants of knowledge of and attitudes toward nutrition during pregnancy. Overall, 69.2% of the participants were unknowledgeable about nutrition. This finding is consistent with a study conducted in the Ambo District, West Shewa Zone, Ethiopia (66.7%).^(35)^ However, this study was higher than the studies conducted in Addis Ababa (26.1%), Southern Ethiopia (38.1%), and East Wollega (35.6).^(33,36,37)^ The results of this study were lower than those of a previous study conducted in Ethiopia, in which 73% of the participants were not knowledgeable about nutrition during pregnancy.^(32)^ This dissimilarity might be due to variations in study years and settings, sociodemographic factors, an individual’s lifestyle, and the accessibility of nutritional information.

In this study, the educational status of pregnant women and accessibility of nutritional information were associated with nutritional knowledge. Accordingly, pregnant women with educational levels of college and above and secondary school were six times more likely to be knowledgeable than those with no formal education. This result is supported by previous findings conducted in Southern Ethiopia, East Wollega, Addis Ababa, and West Shoa Zone, Ethiopia.^(32,35,37)^ This might be because educated women are aware of nutrition and can utilise information delivered through different channels and stakeholders regarding nutrition and health. Similarly, pregnant women who had access to nutritional information were more likely to be knowledgeable than those who had no nutritional information during pregnancy. This result is supported by research conducted in the West Shoa Zone of Ethiopia.^(35)^ This may be because the accessibility of nutritional information has an immediate effect on improving women’s knowledge of nutrition.

Regarding nutritional attitude, 44% of pregnant women had an unfavourable attitude towards nutrition during pregnancy, which was consistent with a previous study conducted in Southern Ethiopia (40.5%).^(37)^ This finding is lower than those of studies conducted in other parts of Ethiopia (68.7%, 66.8%).^(31,37)^ These differences may be due to variations in the study period, sociodemographic characteristics, and individual and social perspectives of the respondents. Educational status was associated with pregnant women’s attitudes toward nutrition; thus, pregnant women with secondary education, college education, and above were more likely to have a favourable attitude regarding nutrition during pregnancy. The finding is supported by the studies conducted in West Shoa and other parts of Ethiopia.^(31,32,37)^ This may be because educated women can understand and utilise nutritional information during their pregnancies. Unlike the findings of previous studies,^(33,34)^ in our study, variables such as pregnant women and husbands’ occupational status, monthly average income of respondents, and family size were not associated with knowledge and attitude towards nutrition. This may be due to the similarity of the population in those variables, and the work environment may not play a role in changing knowledge and attitude unless nutritional information is delivered to the stakeholders.

Overall, this study assessed the knowledge and attitude towards nutrition as well as its determinant factors. These data are essential for providing information on the topic to policymakers, government authorities, and other stakeholders investing in maternal nutrition. This study was conducted in all health centres in the district, and most of the pregnant women in these districts were found in rural areas. This is different from previous studies conducted in urban areas of Ethiopia, which addressed all health centres found in the district, most of which are in rural settings. However, this study has limitations that need to be considered and addressed in further investigations, such as the institution-based cross-sectional study design, which does not address pregnant women not attending antennal services, and the nutritional practices of the respondents, which were not assessed.

### Conclusion and recommendation

In the present study, the knowledge and attitude of the respondents towards nutrition in pregnancy were low compared with previous findings conducted in Ethiopia, especially knowledge about nutrition. The educational level of pregnant women and their access to nutritional information during pregnancy were significantly associated with their knowledge of nutrition. Similarly, the educational level of pregnant women was associated with their attitudes toward nutrition in the study area. Therefore, nutrition education and counselling programmes regarding maternal nutrition are needed to increase maternal nutritional knowledge and attitude, which will improve healthy maternal nutrition tips in the study area through continuous training, home visits, health centre counselling, and other behavioural change communication programmes for nutrition.

## Data Availability

Data will be made available upon request from the corresponding authors.

## Abbreviations

**AOR**: Adjusted odds ratio; **ANC**: Antenatal care; **CI**: Confidence interval; **COR**: Crude odds ratio; **MoH**: Ministry of Health Ethiopia; **SPSS**: Statistical Package for Social Science; **WHO**: World Health Organization; **WU**: Wollega University
